# Field Studies on Immunization of Goat Kids Against Coccidiosis Using X-Radiation-Attenuated Oocysts of Different *Eimeria* Species

**DOI:** 10.3390/vaccines14100843

**Published:** 2026-09-24

**Authors:** Emilio Barba, José Manuel Molina, Otilia Ferrer, Pedro Carlos Lara, José Adrián Molina, Magnolia María Conde-Felipe, Sergio Martín, Carlos Hermosilla, Anja Taubert, Antonio Ruiz

**Affiliations:** 1 Department of Animal Pathology, Faculty of Veterinary Medicine, University of Las Palmas de Gran Canaria, 35416 Arucas, Spain; emiliobarbasan93@gmail.com (E.B.); josemanuel.molina@ulpgc.es (J.M.M.); otilia.ferrer@ulpgc.es (O.F.); jose.molina@ulpgc.es (J.A.M.); sergio.martin@ulpgc.es (S.M.); 2 Medine Department, Fernando Pessoa Canarias University, 35415 Santa María de Guía, Spain; plara@ufpcanarias.es; 3 Parasitology Unit (ParaCOR), Animal Health Department, CRU Zoonoses and Emerging Diseases ENZOEM, University of Córdoba, 14014 Córdoba, Spain; 4 Institute of Parasitology, Faculty of Veterinary Medicine, Justus Liebig University Giessen, 35392 Giessen, Germany; carlos.r.hermosilla@vetmed.uni-giessen.de (C.H.); anja.taubert@vetmed.uni-giessen.de (A.T.)

**Keywords:** goats, immunization, attenuated oocysts, X-radiation, coccidiosis, control

## Abstract

**Background/Objectives**: Caprine coccidiosis caused by *Eimeria* spp. is an important parasitic disease affecting goat health and production, with current control strategies relying mainly on anticoccidial drugs. The emergence of drug resistance and the need for sustainable alternatives highlight the importance of developing effective immunoprophylactic approaches. This study aimed to evaluate the immunoprotective effect of X-ray-attenuated *Eimeria* oocysts against caprine coccidiosis under field conditions and to determine the influence of vaccination on parasite burden, clinical outcomes, and productive performance. **Methods**: A field study was conducted using attenuated oocysts obtained from naturally infected goats from the same farm. The main *Eimeria* species identified were *E. christenseni*, *E. arloingi*, and *E. ninakohlyakimovae*. Three sequential trials were performed to optimize vaccination protocols. Goat kids aged 1–6 weeks received different immunization treatments consisting of doses ranging from 5 × 10^3^ to 6 × 10^4^ sporulated oocysts attenuated by X-ray irradiation at 10–30 kRad (100–300 Gy). Animals were monitored weekly through the evaluation of body weight, faecal consistency, health status, faecal oocyst shedding, and *Eimeria* species composition. **Results**: Vaccination with radiation-attenuated oocysts reduced parasite replication and faecal oocyst shedding, particularly affecting highly pathogenic species such as *E. ninakohlyakimovae*, while maintaining overall *Eimeria* species diversity. Clinical protection was mainly reflected in reduced parasite pressure rather than the complete prevention of mild diarrhoea. Productive parameters were not markedly improved, suggesting that the main benefit of vaccination was improved parasite control and reduced environmental contamination. **Conclusions**: Early administration of X-ray-attenuated *Eimeria* oocysts represents a promising strategy for controlling caprine coccidiosis. Vaccine efficacy depends on appropriate timing and optimization of attenuation conditions. Further studies investigating immune mechanisms, species-specific protection, and performance under commercial farming conditions are required to support the development of sustainable vaccines for goat production.

## 1. Introduction

Goat coccidiosis is a group of parasitic diseases caused by different species of the genus *Eimeria*, causing intestinal parasitosis that may affect goat farms worldwide [1]. Currently, more than 15 species of *Eimeria* affecting goats have been described, and irrespective of age, animals are commonly infected by two, three or even more different *Eimeria* spp. [2,3]. Two species are known to be highly pathogenic, and they are most often the most prevalent, namely *E. ninakohlyakimovae* and *E. arloingi*, while *E. christenseni* and *E. caprina* are considered to have moderate pathogenicity [4,5]. Protozoa included in the genus *Eimeria* are intracellular parasites that colonize the epithelial cells of the intestine, whose main mechanism of pathogenicity is associated with the destruction of host cells during the sexual and asexual reproduction phases of their endogenous cycle [6]. Clinical signs derived from coccidiosis are mainly observed in goat kids and include the elimination of watery feces with traces of yellowish to blackish-brown mucus, which may cause weight loss and dehydration of the animal [7,8]. The costs derived from the use of anticoccidials, together with associated mortality and the decrease in production parameters, are the main causes of the considerable economic losses attributed to coccidiosis in ruminants [9].

The control of goat coccidiosis is based on a combination of management measures [10] and the use of anticoccidials [11]. There are numerous pharmaceutical compounds employed for prophylactic or metaphylactic treatment of goat coccidiosis [12]. However, their use is increasingly limited in most countries and, in addition, reports of anticoccidial resistance are becoming more frequent [13,14,15]. Due to these concerns, a growing interest in the search for alternative control strategies against ruminant coccidiosis has been noticed during the last decade, including immunoprophylactic approaches.

Currently, the poultry industry has many vaccines marketed against avian coccidiosis, which are routinely used on farms. This wide variety includes live vaccines [16], live attenuated vaccines, such as Paracox^®^ (Merck & Co., Ltd., Rahway, NY, USA) and Livacox^®^ (BIOPHARM, Czech Republic) [17,18,19], or vaccines based on the use of less virulent strains of the same parasite, such as NobilisCox ATM^®^ (MSD Animal Health, The Netherlands). The use of non-attenuated live vaccines has a great disadvantage, because the parasites used are totally virulent and can cause symptoms in animals. This fact has determined that in a large part of the world, especially in Europe, their manufacture and commercialization are limited because they are currently unlicensed. To solve this problem, a second line of live attenuated vaccines was developed from the passage of the parasite through embryonated chicks [20], generating a vaccine that replicates at lower levels with very high safety and efficacy, whose only drawback is the high production price.

Many recombinant DNA vaccines addressed to different poultry *Eimeria* species have been developed worldwide, although they are not currently commercially available and are therefore not a viable alternative in the short term [16,21]. Numerous proteins from different *Eimeria* species have been identified that could act as immunogens. The most widely studied are proteins secreted from micronemes and organelles located in the apical region of the parasite [22], but also gametocyte proteins [23,24], and others such as S07 secreted by *E. tenella* sporozoites [25,26] and 3-1E found in *E. acervulina* merozoites [27,28]. All these antigens induce a variable degree of protection in homologous infections, but only a few cases have demonstrated cross-immunity among different *Eimeria* species. Among those showing promising results are profilin [29], EF-1a [30], and SO7 [31]. In general, as a prerequisite, standardization of the protocols used for the assessment of vaccine efficacy is needed to make comparative evaluations and validations [32].

Immunizations with live or attenuated forms of coccidia have been poorly addressed in other domestic species such as ruminants [33,34], despite the fact that numerous studies have shown that ruminant *Eimeria* species can generate specific protection after primary infections with the parasite, in which Th-1 type responses are predominant [35]. In this line of research, Ruiz et al. [36] have demonstrated that the immunization of goat kids with X-ray attenuated oocysts determines protective immune responses against coccidiosis produced by *E. ninakohlyakimovae*. The immunization protocol was based on methods like those previously described for the attenuation of oocysts in avian vaccines [37] and showed that the immunized goat kids had no apparent symptoms of disease during the course of the infection, and shed fewer oocysts than animals infected with non-attenuated oocysts. In addition, after challenge with non-attenuated oocysts, the immunized animals had reduced faecal oocyst counts and a general improvement in the clinical presentation of coccidiosis, similar to that obtained in goat kids primarily and subsequently challenge-infected with non-attenuated oocysts [36]. However, given that monospecific infections hardly occur in the field and that immunity against *Eimeria* species predominantly seems to be species-specific, an attempt to develop a vaccine including several caprine *Eimeria* species, particularly the most pathogenic ones, has recently been made by the same research group following a similar methodology [38]. The results of this study indicate that immunoprotection was less evident than that reported for monospecific vaccination against *Eimeria ninakohlyakimovae*. Therefore, further improvement of the immunization protocol is needed.

In the present study, we have performed a field study on the optimization of immunizing protocols against goat coccidiosis by using X-ray attenuated oocysts of a mixture of *Eimeria* species, including the most pathogenic ones (*E. ninakohlyakimovae*, *E. arloingi* and *E. christenseni*). For this purpose, factors such as the age of immunisation of the animals, the degree of attenuation of the oocysts or the dose of immunogen applied have been considered.

## 2. Materials and Methods

### 2.1. Recovery and Purification of Eimeria spp. Oocysts for Immunogen Preparation

Faecal samples were collected from naturally infected animals from the same farm where the experiment was to be carried out. As the percentage of the different *Eimeria* species varied according to the age of the animals, samples were collected from several open pens (usually 3–4 pens), making sure that they belonged to young animals (less than 4 months old) in order to approximate, as closely as possible, the age at which goat kids are most susceptible to clinical coccidiosis.

Routinely, approximately 1 kg of faecal material was collected from each pen. Just after arrival at the laboratory, faecal oocysts were purified following the method described by Hermosilla et al. [39]. Briefly, faecal material was washed with tap water and filtered with decreasing pore sizes to reduce detritus. The resulting suspension was dispensed in uncovered plastic receptacles containing saturated sugar solution at a 1.5 g/L density. The containers were filled to form a meniscus and covered with glass plates to collect oocysts by flotation. Floating oocysts were transferred to a tray by washing them off the glass plates and then to a flask. To increase the yield, the mixture was stirred every two hours, and the glass plates were replaced, for a total of 3 consecutive days. All washes were kept at 4 °C and concentrated by centrifugation at 1500× *g* for 10 min. Oocyst sporulation was achieved by incubation at room temperature (22 °C) for one week in 2% potassium dichromate (Panreac, Barcelona, Spain) solution under constant stirring and air infusion into the suspension.

The number of oocysts present in the different pen batches was determined by a modified McMaster method [40], but using 1 mL of oocyst suspension instead of 1 g. The proportion of *Eimeria* species in each batch was established under an Olympus microscope (Olympus CH40, Tokyo, Japan) using a 40× objective and based on morphometric criteria [41,42,43]. The final inoculum used as immunogen was prepared by mixing oocysts from the different batches, and care was taken to ensure that the final inoculum contained an equal proportion (1/3) of the three most frequent *Eimeria* species in the farms: *E. ninakohlyakimovae*, *E. arloingi* and *E. christenseni*.

All these procedures were exactly repeated in the same way on three occasions over a whole period of 2 years, with the aim of having fresh oocysts for the three corresponding experiments included in the present study. In all cases, final oocyst suspensions were kept at 4 °C until further use.

### 2.2. Attenuation of Eimeria spp. Oocysts

Prior to the attenuation of the oocysts, they were washed three times with sterile distilled water (1500× *g*, 10 min) to eliminate the potassium dichromate. They were suspended in sterile distilled water and finally divided into culture flasks of 25 cm^2^ (Nunc) at a rate of 20 mL per flask. Depending on the experiment, flasks were subjected to a total irradiation of 10, 20 or 30 kRad (100, 200 or 300 Gy). The ionizing radiation was performed at 6 mV of X-rays and a speed of 50 cGy/min in a Mevatron^®^ linear accelerator (Siemens, Berlin, Germany) owned by the Hospital Universitario de Gran Canaria Dr. Negrín (Las Palmas, Gran Canaria, Spain). Compared to non-attenuated oocysts, different degrees of irradiation of *Eimeria ninakohyakimovae* sporozoites translate into distinct developmental capacities in *in vitro* culture systems (unpublished data; manuscript currently under peer review).

### 2.3. Animals and Farm Study

The experiment was carried out on a farm located in the hamlet of Bahía Feliz, which belongs to the municipality of San Bartolomé de Tirajana on the island of Gran Canaria (Spain), having the following coordinates: 27°47′29.4″ N 15°31′15.0″ W. The farm was located in an arid area with a dry climate and low rainfall, with an average temperature of 21 °C, reaching its maximum in August (28 °C) and minimum in January (15 °C). It is a goat production farm, whose main profits are derived from the sale of cheese made from goat’s milk, as well as the sale of goat kids for meat.

The approximate census of the farm at the time of the study was 5030 goat heads, which were divided into different pens. A total of 3740 animals were breeding females following a semi-extensive production model, with one milking per day. The rest of the animals were divided into 90 breeding males, 650 non-breeding females aged between 4 and 12 months, 300 non-breeding females under 4 months and 250 fattening males, all under intensive production. The farm also had a census of approximately 835 sheep, which were located next to the goats: 540 breeding females, 35 breeding males, 200 non-breeding animals aged between 4 and 12 months, and 60 non-breeding animals less than 4 months old.

The goats reared on this farm belong to the Majorera breed, a breed that is very well adapted to arid and semi-arid climates [44]. Majorera goats are short-haired and have large heads and arched horns. Being native to the island of Fuerteventura, it is currently the breed with the largest existing population in the Canary Islands. In terms of production, it is considered a breed of very good dairy aptitude, able to provide a large yield of milk with a high protein and fat content, mostly used for cheese production [45].

There are usually three kidding seasons per year on the farm: February–March, June–July and October–November, with the first and the last having the largest number of animals. The goat kids are separated from their mothers at birth, given colostrum for two days and manually fed with milk replacer (Bacilactol, Capisa^®^, Las Palmas de Gran Canaria, Spain) for a week. Afterwards, the animals are fed with a semi-automatic artificial lactation system twice a day. From the first week of life onwards, hay is provided *ad libitum*, and from the first month, starter concentrate (Capisa^®^, Spain) is also provided. Artificial feeding is carried out in groups of approximately 25 animals so that each animal has its own nipple-shaped rubber tip, disinfected between groups with Povidone Iodine to avoid infections. When the animals are two months old, lactation is reduced to once a day for at least 15 days, at which age the animals are completely weaned. All the females are destined for restocking while the males are for fattening, with some of the males destined for reproduction coming from external farms to avoid inbreeding.

### 2.4. Experimental Design

A total of 3 experiments were performed sequentially with protocols established based on immunoprotection results obtained in previous assays, so they should not be interpreted as independent efficacy trials but regarded as being part of a sequential optimization study. The three experiments were carried out over a period of two years, during which no substantial changes in the management of the animals on the farm were noticed. The justification for the number of animals per experimental group was based on (1) the availability of newborn kids in the kidding season when the different trials were scheduled, and (2) the total number of animals required for each trial, which in turn varied depending on the number of experimental groups per trial.

#### 2.4.1. First Trial

A total of 163 female goat kids were divided into 4 age groups according to the time at which they were to be immunized (week 3, 4, 5 and 6 of life). In turn, several subgroups (*n* = 6–7 animals) were established according to the infective dose (5 × 10^3^, 1 × 10^4^ or 2 × 10^4^ attenuated *Eimeria* spp. oocysts) and the degree of irradiation used for attenuation (10 or 20 kRad). In addition, a control group consisting of animals not immunized with irradiated oocysts was included.

For an easier reference to experimental groups in the result and discussion sections, the following arbitrary nomenclature was followed for immunization at the different ages: 5M-10K (immunization with 5 × 10^3^ oocysts attenuated at 10 kRad); 5M-20K (immunization with 5 × 10^3^ oocysts attenuated at 20 kRad); 10M-10K (immunization with 1 × 10^4^ oocysts attenuated at 10 kRad); 10M-20K (immunization with 1 × 10^4^ oocysts attenuated at 20 kRad); 20M-10K (immunization with 2 × 10^4^ oocysts attenuated at 10 kRad) and 20M-20K (immunization with 2 × 10^4^ oocysts attenuated at 20 kRad) (Table 1).

#### 2.4.2. Second Trial

Based on the results of the First Trial, the animals of the second trial (*n* = 96) were divided into two age groups, depending on the time of inoculation (1 and 2 weeks of age). Within each age group, different subgroups (*n* = 9–10 animals) were established depending on the infecting dose (2 × 10^4^ or 6 × 10^4^ *Eimeria* spp. oocysts) and the degree of irradiation used for oocyst attenuation (10 or 20 KRad). A negative non-immunized control group was also included, which was maintained under the same conditions as the study groups (*n* = 15).

As mentioned above, for an easier reference to experimental groups the following arbitrary nomenclature was used: 20M-10K (immunization with 2 × 10^4^ oocysts attenuated at 10 kRad); 20M-20K (immunization with 2 × 10^4^ oocysts attenuated at 20 kRad); 60M-10K (immunization with 6 × 10^4^ oocysts attenuated at 10 kRad) and 60M-20K (immunization with 6 × 10^4^ oocysts attenuated at 20 kRad) (Table 1).

#### 2.4.3. Third Trial

Finally, in the third trial, all animals were immunized at week two of life. Three study groups (*n* = 12) were established. The first group was inoculated with 2 × 10^4^ *Eimeria* spp. oocysts attenuated at 20 kRad (20M-20K). The other two study groups were immunized with *Eimeria* spp. oocysts attenuated at 30 kRad, with the number of oocysts inoculated being 2 × 10^4^ (20M-30K) or 6 × 10^4^ (60M-30K). As in the previous two trials, a non-immunized negative control group was considered (Table 1).

All goat kids selected for the whole study were conveniently identified during the first days of life with numbered plastic tags attached to a chain around their necks. Only healthy animals were selected, and all groups were of similar average weight. Experimental animals were reared in the same facilities as the rest of the goat kids on the farm and were raised following the same management practices. No anticoccidial treatment was administered to the goat kids included in the experiment.

The effect of immunization with X-irradiated oocysts was evaluated upon natural challenge infections within the rearing environment. For this purpose, immunoprotection was assessed weekly from the time of immunization until the 14th week of life by productive (body weight), clinical (presence or absence of clinical symptoms) and parasitic (faecal oocyst counts) parameters. Therefore, goat kids from each experimental group were weighed weekly, examined for the presence of clinical symptoms, and faecal samples were individually collected from the rectum. Faeces were weighed and stored in 2% potassium dichromate at 4 °C until processed.

All animal procedures were conducted in strict accordance with national ethics, the current European Animal Welfare Legislation (ART13TFEU) and with institutional review board-approved protocols (project OEBA-ULPGC 16/2018 authorized on 30 November 2018, administrative decision 902/2018, Department of Agriculture and Livestock, Government of the Canary Islands).

### 2.5. Parasitological Analysis

Individual faecal samples were analyzed by a modified McMaster method [40] to quantify the excretion levels of *Eimeria* spp. oocysts. Routinely, one gram of faeces was mixed with 30 mL of a saturated NaCl solution (1.2 g/mL density) and analyzed in a McMaster chamber. However, in cases of limited faecal material (mainly in very young goat kids or animals showing diarrhea), the following procedure was followed: 30 mL of saturated NaCl solution was used for samples weighing more than 0.4 g, 10 mL was used for weights between 0.2 and 0.4 g and 5 mL was used for weights below 0.2 g. If necessary, in the presence of a very high number of oocysts in faecal samples, a dilution of the samples was carried out (from 10 to 100 times) to perform an adequate count [46]. By considering the dilutions and the weight of the faecal samples, the results of the counting were expressed as oocysts per gram of feces (OPG). Attenuated oocysts and oocysts coming from vaccinated animals showed the same morphological characteristics as non-attenuated ones. Therefore, counts could include oocysts from both immunizations and natural infections on the farm.

Once the count was done, the percentage of each of the *Eimeria* species present in the sample was determined by following keys previously reported [41,42,43]. The analysis was performed under a 40× objective on an Olympus microscope. For this purpose, part of the McMaster solution was transferred to a homemade chamber consisting of two 1/3 slides (22 × 22 mm) stacked to the edge of a full 22 × 66 mm slide. For the observation of the oocysts, the open space in the center of the chamber was filled with the oocyst solution to form a meniscus and then covered with a 22 × 40 mm cover slide. Routinely, to determine the species for each experimental group and sampling point, when possible, a total of 20 sporulated oocysts from all the animals in that group were analyzed. Accordingly, the total number of oocysts analyzed per group ranged from 120 oocysts (for groups with *n* = 6) to 240 oocysts (for groups with *n* = 12).

### 2.6. Clinical and Production Determinations

Faecal consistency (score) was evaluated by the same operator immediately after collecting the individual faecal samples, using for this purpose the following scores [8,36]: (1) normal formed faeces; (2) faeces with slightly reduced consistency; (3) faeces with moderate reduction in consistency (4) watery diarrhoea (5) explosive watery diarrhea containing traces of blood and/or mucus. The presence of faecal material in the perianal region (soiling) was also evaluated with the following scores: (1) no soiling, (2) dry soiling and (3) wet soiling.

The presence of other clinical signs compatible with coccidiosis was also registered, such as anorexia, dehydration, weakness or recumbency. Additionally, the occurrence of respiratory symptoms, contagious ecthyma lesions or evidence of other common pathologies in goat kids around that age were also monitored weekly. As for the production effect of immunization, the weight of the animals was recorded within the same intervals.

### 2.7. Statistical Analysis

For the statistical analysis, faecal oocyst counts, expressed as oocysts per gram (OPG), were logarithmically transformed as log(OPG + 1). Body weight and faecal consistency were also assessed sequentially in all animals throughout the study period. As these parameters, together with OPG, were measured repeatedly over time in the same animals, longitudinal data were analyzed using a repeated-measures General Linear Model.

For Trial 1, longitudinal OPG data were analyzed using a mixed-effects model, with immunization cohort, treatment, time, and their relevant interactions included as fixed effects and animal included as a random effect to account for repeated measurements within individuals. The four immunization-age cohorts were compared with the same non-immunized control group, which was included as a common comparator throughout Trial 1. Planned comparisons between each vaccinated cohort and the common control were adjusted for multiplicity using Dunnett’s procedure. The same statistical approach was applied to Trial 2, as the immunization was evaluated at two different ages. By contrast, in Trial 3, immunization was evaluated at a single age (2 weeks of life), and a common non-immunized control group was established for comparison with each of the three experimental groups.

The relative reduction in oocyst excretion was calculated as (C − T)/C × 100, where C represents the mean OPG of the non-immunized control group and T represents the mean OPG of the corresponding vaccinated group [47]. Overall treatment efficacy was additionally estimated from the area under the OPG-versus-time curve (AUC), calculated for each experimental animal. Body-weight gain over time was expressed as growth rate, calculated as (ln weight 2 − ln weight 1)/t × 100, where t represents the number of days between sampling time points 1 and 2.

Differences in *Eimeria* species distribution and soiling among groups were analyzed using a non-parametric chi-square test. Statistical analyses were performed using SigmaPlot 14.5 (Systat Software Inc., Erkrath, Germany), and differences were considered statistically significant at *p* < 0.05.

## 3. Results

### 3.1. Oocyst Counts

#### 3.1.1. First Trial

Animals immunized at 3 weeks of age (Figure 1A) showed faecal oocyst excretion levels close to zero at the time of immunization. OPG values increased progressively thereafter, reaching maximum levels at week 5 of life in groups immunized with different doses of oocysts attenuated at 10 kRad, and at week 6 in those immunized with oocysts attenuated at 20 kRad, regardless of the infective dose administered. In groups immunized at weeks 4, 5, and 6 of life (Figure 1B, Figure 1C and Figure 1D, respectively), the dynamics of oocyst shedding were very similar to those observed in animals immunized at week 3. Mean maximum OPG values were reached between the 5th and 6th weeks of life, irrespective of both the oocyst dose and the attenuation level. Following the peak in oocyst excretion, a progressive decline in the number of oocysts shed was observed in all groups, with the lowest mean value at the end of the experiment (week 14 of life) being 5840.2 ± 4218.6 OPG (group 5M-10K).

The non-immunized control group was the same throughout the First Trial and displayed an oocyst shedding pattern comparable to that observed in the experimental groups (Figure 1A–D). Maximum oocyst excretion levels were reached at approximately weeks 5–6 of life and subsequently declined until the end of the experiment. Overall, oocyst counts showed high variability among animals within the same group, as reflected by the large standard errors recorded. In some animals, OPG values reached nearly 6 million at week 5 of life.

When comparing mean OPG values between the experimental and control groups, a certain trend towards a reduction of oocyst shedding was observed, with the most marked reductions occurring in groups 10M-20K and 20M-20K (animals immunized at 3 weeks of age). This reduction was particularly evident between weeks 5 and 6, coinciding with the period of maximum oocyst shedding. In general, differences between immunized and control groups became less apparent when immunization was performed at 5 or 6 weeks of age. However, no statistically significant differences were detected in any comparison.

To further assess the effect of immunization on oocyst excretion, the area under the curve (AUC) was calculated, and the percentage reduction in OPG values relative to the control group was determined (Figure S1A–D). The greatest reductions in OPG were observed in groups 20M-20K (~60%) and 10M-20K (~40%) following immunization at 3 weeks of age. The reduction in OPG was less pronounced when attenuated oocysts were administered at 4 weeks of age, while little to no reduction was observed when immunization was performed at 5 or 6 weeks of age. This trend was also observed when analyzing the AUC from weeks 8 to 14 of life, corresponding to a period of at least two weeks after immunization of the oldest animals (i.e., those immunized at 6 weeks after birth) (Figure S1D).

#### 3.1.2. Second Trial

Animals immunized during the first and second weeks of life showed oocyst counts close to zero from the time of immunization until week 4 of life (Figure 2A,B). From this point onwards, OPG values progressively increased, reaching their maximum levels at week 6 in all groups. One week after the OPG peak, a reduction in oocyst shedding was observed in most study groups, followed by a moderate increase during the subsequent week. At the time corresponding to the peak oocyst shedding observed in the control group, all immunized groups showed lower oocyst excretion, with the most pronounced reduction occurring in the 60M-10K group (*p* < 0.01) in animals immunized during both the first and second weeks of life. Similarly, OPG counts at the end of the experiment (weeks 7 and 8 of life) were lower in immunized goat kids than in control animals in most groups, with statistically significant differences ranging from *p* < 0.05 to *p* < 0.01 depending on the group. Consistently, the area under the curve (AUC) was lower in all immunized groups compared with control animals (Figure S2A,B), with the greatest reduction observed in the 60M-10K group when immunization was performed at 2 weeks of age (73%).

#### 3.1.3. Third Trial

Goat kids from the Third Trial showed OPG counts close to zero during the first two sampling points, corresponding to weeks 2 and 3 of life (Figure 3). From this point onwards, oocyst shedding progressively increased, reaching a peak in week 6 of life in both immunized and non-immunized groups. At the time of maximum oocyst shedding, OPG counts were lower in immunized groups than in the control group. A similar trend was observed at the final sampling point (week 8 of life), with the immunized groups showing reduced OPG values compared with controls. Statistically significant differences were detected for the 20M-20K immunized group (*p* < 0.05). Consistent with the lower OPG values, immunized groups showed a reduction in the overall area under the curve (AUC), ranging from approximately 10 to 25% (Figure S3).

### 3.2. Eimeria Species

The frequency of the different *Eimeria* species varied considerably with age throughout the study across the different experimental groups and trials. However, no consistent differences were observed that could be associated with a particular immunization strategy and the prevalence of any specific species. Figure 4 illustrates the temporal variation in the three *Eimeria* species considered the most pathogenic for goats, together with the remaining less pathogenic species, in both immunized animals and non-immunized controls. The results from the three experiments were analyzed together, with all immunized animals grouped regardless of the immunization strategy applied.

Overall, the most prevalent species throughout the study, in both immunized and control animals, was *E. christenseni* (Figure 4A). Its prevalence ranged from approximately 50% to 80%, with maximum values observed around week 6 of life. The second most prevalent species was *E. arloingi*, with frequencies ranging from approximately 10% to 45% (Figure 4B). The highest frequency of this species was observed at week 2 of life, although relatively high levels were also detected at the final sampling point (week 14 post-infection). *E. ninakohlyakimovae* was detected at moderate to low frequencies throughout the study, with peak values observed at week 4 post-infection and at the end of the experiment (week 14 post-infection) (Figure 4C). Finally, the combined prevalence of the remaining *Eimeria* species was relatively low, at approximately 5%, with a slight increase during the final week of the study (Figure 4D).

When comparing the prevalence of the different *Eimeria* species between immunized and non-immunized animals, a broadly similar pattern was observed, although some differences were detected. The area under the curve (AUC) for *E. christenseni* prevalence was slightly higher in immunized animals (+5.2%), whereas the AUC for the group of “other *Eimeria* species” was essentially unchanged (−1.6%). In contrast, a reduction in prevalence was observed for *E. arloingi* and *E. ninakohlyakimovae*, with decreases of 6.0% and 31.7%, respectively, in immunized animals.

The specific weekly fluctuations in the prevalence of *E. christenseni*, *E. arloingi*, *E. ninakohlyakimovae*, and the group of “other *Eimeria* species” according to the different immunization schedules are presented in Figures S4–S10.

### 3.3. Clinical Parameters

#### 3.3.1. Faecal Consistency

No specific pattern was found when analyzing the faecal score across the different experimental groups for each week of immunization, so the evaluation of this parameter was conducted from a global, rather than group-specific, perspective by comparing between immunized and control animals.

The overall mean for the whole experiment was 1.39 in the first trial, approximately the same in the second trial (1.36) and slightly higher in the third trial (1.55). Accordingly, more than 50% of the animals had normal faeces (score 1) in the three experiments (Figure 5), around 25% had score 2 and less than 10% of goat kids showed faecal scores 3 or 4 (Figure 5). Although some variations were observed, no significant differences were found between the control group and the immunized animals in any of the four weeks of immunization of Trial 1 (Figure 5A) when considering the five score categories separately, and the same was observed in Trial 3 (Figure 5C). However, immunized animals of the second trial had a markedly lower frequency of score 2 than the control group (Figure 5B).

When the overall score was considered, animals immunized at weeks 4, 5 and 6 had lower faecal scores than control goat kids in Trial 1, with significant differences being recorded in the fifth week of immunization (*p* < 0.05) (Figure S11A). On the other hand, groups immunized at the second week of both Trial 2 and 3 had higher faecal scores than controls, with significant differences being recorded in Trial 2 (*p* < 0.05) (Figure S11B).

During weeks 3, 4 and 5 of life, animals had the highest values of the faecal score in Trial 1. Those values decreased in weeks 7 and 8 and slightly increased afterwards around the end of the study (Figure S12A). Accordingly, faecal consistency in Trial 2 gradually decreased from the first week up to the end of the trial (week 8 of life) (Figure S12B). However, weekly variation in faecal scores was more regular in Trial 3, apart from week 3, where significantly lower values were found compared to most of the sampled weeks (Figure S12C). This was the only experiment in which differences between the immunized and control groups were found regarding weekly variation in stool consistency. Thus, significantly lower consistency (*p* < 0.05) was found in week 5 of life in immunized animals, while the opposite was recorded in week 7 (*p* < 0.01) (Figure S12C). Finally, when assessing the effectiveness of the different formulations of the immunogen (infection dose/radiation intensity), the overall faecal consistency was only reduced in group 10M-20K (*p* < 0.05) (Figure S13A).

#### 3.3.2. Soiling

Similarly to stool consistency, more than 50% of the animals did not show soiling during the study; dry soiling was observed in about 30% of the goat kids, and less than 10% of them displayed humid soiling (Figure 6). Approximately the same global results were recorded in the three experiments. Variations in soiling scores were observed between immunized and control animals according to the week of immunization. Thus, goat kids immunized in week 5 or 6 of life exhibited less dry and humid soiling than controls (Figure 6A), while the effect of the immunization over this parameter was not consistent when the immunogen was administered earlier in time (weeks 1 or 2 of life) (Figure 6B,C).

Taking into account the different formulations of the immunogen, the effect of immunization on soiling was less evident (Figure S14). The only positive impact was observed when the percentage of humid soiling was assessed, with some experimental groups showing a certain tendency to have lower value than non-immunized controls (e.g., 5M-10K, 5M-20K, 10M-20K and 20M-10K in Trial 1; 20M-10K in Trial 2; 20M-20K; 20M-30K and 60M-30K in Trial 3).

### 3.4. Production Parameters

#### 3.4.1. Body Weight

The evolution of body weight in the experimental groups was highly similar across the three trials. Body weight progressively increased from approximately 4–5 kg at the beginning of the experiment to around 8–9 kg at the end of Trials 2 and 3 (final sampling at week 8 of life), and to approximately 13 kg at the end of Trial 1 (week 14 of life) (Figure S15). Differences between control and immunized animals were detected only in a limited number of cases, such as in the 20M-20K group (Trial 1, immunization at week 5 of life), where a significant difference was observed (*p* < 0.05).

#### 3.4.2. Weight Gain

Despite minor variations among groups, overall growth rates followed a very similar pattern across the three trials (Figure 7A–C). Three main growth peaks were identified: between weeks 2 and 3, between weeks 5 and 6, and, most prominently, between weeks 8 and 9 of life. Due to differences in the experimental design among trials, the first peak was clearly observed only in Trial 1, whereas the third peak was only detected in Trial 3. The highest growth rate was recorded at week 9 of life in Trial 1 in the control group (2.61 ± 0.10%) (Figure 7A). In contrast, the lowest values (close to zero) were observed at week 8 of life in both immunized and control groups (Figure 7C).

When comparing overall growth rates between immunized animals and controls in Trial 1, significantly higher growth rates were observed in the control group at week 9 of life (*p* < 0.05) (Figure 7A). In Trial 2, consistently higher growth rates were observed in immunized groups during weeks 6, 7, and 8 of life, with significant differences detected only at the final sampling point (*p* < 0.05) (Figure 7B). Conversely, growth rates were highly similar between immunized and control animals throughout all sampling points in Trial 3 (Figure 7C).

The analysis of growth rates according to specific immunized groups showed that no significant differences were detected in Trials 1 and 2 (Figure S16A,B). In Trial 3, only the 20M-20K group tended to have higher overall growth rates than the control group, although this difference was not statistically significant. Nevertheless, a significant difference was observed in this same group when growth rate was calculated between weeks 4 and 5 of life (*p* < 0.05).

Finally, when considering all experimental groups and sampling points, overall growth rates ranged from 1.30 ± 0.08 to 1.46 ± 0.04, with no significant differences detected either among the three trials or between immunized and control animals.

## 4. Discussion

The present study evaluated the efficacy of a multispecies vaccine based on radiation-attenuated *Eimeria* oocysts for the control of caprine coccidiosis under field conditions. The results demonstrate that vaccination can significantly reduce parasite replication, as evidenced by lower oocyst shedding and cumulative oocyst output, particularly when administered during the first weeks of life. Although effects on clinical signs and productive performance were limited, the reduction in parasite excretion provides strong evidence that immunization with attenuated *Eimeria* oocysts can contribute to the control of caprine coccidiosis by reducing environmental contamination and transmission within herds.

The protective effect observed in this study is consistent with previous reports demonstrating the feasibility of using irradiated *Eimeria* oocysts as live attenuated vaccines. In poultry, gamma-irradiated oocysts have been shown to induce protective immunity while maintaining sufficient parasite viability to stimulate effective immune responses [48,49,50]. Comparable studies in ruminants remain limited. However, previous work from our research group demonstrated that vaccination of goat kids with irradiated *Eimeria ninakohlyakimovae* oocysts [36], as well as a multispecies irradiated oocyst preparation under experimental conditions [38], significantly reduced oocyst shedding and disease severity following challenge infection. The present study extends these findings by providing, to our knowledge, the first evidence that a multispecies vaccine based on irradiated *Eimeria* oocysts can confer measurable protection against naturally acquired caprine coccidiosis under field conditions.

The reduction in oocyst shedding represented the clearest indicator of vaccine efficacy. Although vaccinated and control animals followed a similar age-related pattern of oocyst excretion, with peak shedding occurring between five and six weeks of age, vaccinated kids consistently exhibited lower OPG values and reduced cumulative oocyst output. The greatest reductions were observed when vaccination was administered during the first two weeks of life, resulting in decreases of up to 73% in the area under the oocyst excretion curve. In contrast, vaccination at five or six weeks of age produced only minimal effects. Upon detailed analysis of the associated immune response, these findings highlight the importance of synchronizing vaccination with the epidemiology of infection, allowing protective immunity to develop before the period of maximum parasite amplification.

Host age appears to be a critical determinant of vaccine efficacy. Increasing evidence indicates that the development of protective immunity against protozoan and helminth parasites is strongly influenced by the maturation of the host immune system [51,52,53]. In goats, Ruiz et al. [54] reported that young kids are considerably more susceptible to clinical coccidiosis than adults, emphasizing the role of age-dependent immune competence in disease outcome. Likewise, Matos et al. [55] showed that kids infected with *E. ninakohlyakimovae* at 3–5 weeks of age developed greater protection upon reinfection than animals first infected at seven weeks of age. The superior efficacy of early vaccination observed in the present study is likely related both to host immune development and to the biology of live attenuated vaccines. Protection induced by attenuated *Eimeria* vaccines depends on limited endogenous parasite development, antigen presentation, and activation of cell-mediated immune responses before challenge exposure [56]. Consequently, vaccination administered after natural exposure has already occurred may be less effective because parasite replication has already been initiated. Similar mechanisms have been described in poultry, where attenuated vaccines induce protection through controlled parasite development while minimizing intestinal damage [57,58]. Together, these observations suggest that vaccination should be implemented during the earliest weeks of life, before substantial natural exposure occurs.

The magnitude of protection was also influenced by vaccine formulation, particularly the infective dose and the degree of irradiation. Jenkins et al. [37] demonstrated in poultry that effective immunoprotection depends on achieving an appropriate balance between parasite viability and attenuation. Our findings support this concept, as differences among vaccine formulations resulted in variable levels of protection. The best overall efficacy was consistently associated with an infection dose of 20,000 oocysts irradiated at 20 kRad. The irradiation dose itself represents a critical determinant of vaccine performance. Radiation attenuation preserves a broad antigenic repertoire while restricting parasite development, but excessive attenuation may reduce antigen exposure and immunogenicity, whereas insufficient attenuation may compromise vaccine safety [20,59]. Accordingly, previous studies in chickens reported that sporulated oocysts irradiated at 15–20 kRad induced substantial protection while producing only minimal oocyst shedding after vaccination, whereas lower irradiation doses provided inferior protection [37]. Our results closely parallel these observations, with 20 kRad consistently producing superior parasitological outcomes compared with 10 kRad. However, the optimal irradiation level may vary among host–parasite systems. For example, Thabet et al. [60] reported that *Eimeria tenella* oocysts irradiated at 50 kRad induced greater protection in chickens than those irradiated at lower doses, while maintaining normal health and productive performance. Collectively, these findings indicate that irradiation protocols should be optimized empirically for each *Eimeria* species and host species in order to balance safety, parasite viability, and immunogenicity.

Although vaccination markedly reduced parasite output, protection was not complete and considerable individual variation was observed. Such variability is expected in coccidian infections because host susceptibility, immune responsiveness, intestinal environment, and parasite replication dynamics differ among individuals. Increasing the number of animals per group could have helped to account for this variability and improve the statistical power to detect differences in OPG counts, and potentially in clinical and production parameters. Although the variability in absolute OPG values and the insufficient statistical power represent limitations of the study, the consistent reduction in cumulative oocyst shedding indicates that vaccination effectively limited parasite amplification despite this variability. Importantly, complete sterilizing immunity is rarely the objective of *Eimeria* vaccination. As observed in other livestock species, the principal goal is to reduce parasite replication and environmental contamination rather than to prevent infection entirely [56,61]. Consequently, even partial reductions in oocyst excretion may have important epidemiological consequences by lowering infection pressure and transmission within herds.

Vaccination reduced parasite burden without substantially altering the structure of the *Eimeria* community. Among the identified species, *Eimeria christenseni* was predominant throughout the study, reaching prevalence values of approximately 50–80% and peaking around six weeks of age. This finding agrees with previous reports describing *E. christenseni* as one of the most prevalent *Eimeria* species in young goats and an important contributor to caprine coccidiosis [33,62]. Its persistence in vaccinated animals indicates that vaccination did not prevent establishment of infection but rather reduced overall parasite replication, as described in other live attenuated vaccines [57,61]. Some species-specific effects were nevertheless observed. Vaccinated animals showed lower frequencies of *E. arloingi* and particularly *E. ninakohlyakimovae*, with reductions in cumulative prevalence of approximately 6% and 32%, respectively. These differences may reflect variation among species in tissue tropism, reproductive capacity, endogenous development, and antigenic composition [56,59]. The reduction of *E. ninakohlyakimovae* is especially relevant because this species is considered highly pathogenic and is frequently associated with severe intestinal lesions and clinical disease in goats [33]. Despite these differences, the overall age-related succession of *Eimeria* species was preserved, indicating that vaccination reduced infection intensity while allowing the natural acquisition of immunity against the broader parasite community.

Despite the clear reduction in parasite excretion, the effects on clinical parameters were limited. Most animals maintained normal faecal consistency, and vaccination did not consistently influence the occurrence of diarrhea or perineal soiling. This probably reflects the relatively low clinical expression of infection under the conditions of the study, where parasite exposure may not have been sufficient to induce severe intestinal damage. Clinical disease is primarily associated with epithelial destruction caused by endogenous developmental stages rather than with oocyst shedding itself [33,63], particularly if the predominant *Eimeria* species are of low pathogenicity. In this regard, our research group has demonstrated that the GC strain of *E. christenseni*, isolated specifically from the farm that is the subject of this study, exhibited low pathogenicity [64]. In general, reductions in parasite replication and oocyst shedding may not necessarily be accompanied by measurable changes in clinical indicators when infection pressure is moderate or if the *Eimeria* species involved are mostly nonpathogenic.

Similarly, no consistent improvements in body weight gain or growth performance were detected. Productive losses associated with coccidiosis generally occur when severe intestinal damage impairs nutrient absorption and feed efficiency [33,63]. The limited clinical impact observed in the present study likely reduced the possibility of detecting growth-related benefits. Importantly, however, vaccination did not adversely affect performance in the parameters analyzed here, supporting the safety of the attenuated vaccine approach. Under field conditions, the principal benefit of vaccination may therefore reside in long-term reductions in environmental contamination and infection pressure rather than in immediate improvements in individual productivity [58].

Although immunization with attenuated oocysts used here only led to a limited improvement in body weights and growth rates, this vaccination approach could have beneficial outcomes beyond the immediate production performance of the animals. By reducing oocyst release, vaccination could contribute to reducing environmental contamination, thereby decreasing the exposure pressure to coccidial infections in goat kids from subsequent farrowing seasons. This could help to mitigate the cumulative impact of coccidiosis on herd productivity over the long term. Additionally, by reducing the prevalence of coccidiosis and subsequent use of anticoccidials, the selective pressure for antiparasitic drugs could be minimized. Therefore, the economic impact of the vaccine could be derived not only from the improvement of individual growth rates, but also from the possibility of long-term control of the disease and a more sustainable management of the farm.

Unlike the situation in small ruminants, live anticoccidial vaccines have been successfully used in the poultry industry for several decades. Nevertheless, their widespread adoption has been constrained by the complexity and cost of production. One of the principal challenges is the need to include multiple *Eimeria* species within a single formulation because protective immunity is largely species-specific. Differences among species in pathogenicity, immunogenicity, and epidemiology further complicate vaccine standardization and direct comparisons among formulations [32]. Similar challenges are likely to affect vaccine development for goats. Moreover, large-scale production of live vaccines requires substantial numbers of viable oocysts, making vaccine manufacture labor-intensive and expensive. Future research should therefore focus on identifying the minimum immunizing dose capable of inducing robust protection, optimizing irradiation protocols, and improving production efficiency to enhance the economic feasibility and scalability of commercial vaccine development.

## 5. Conclusions

In conclusion, the present study demonstrates that radiation-attenuated multispecies *Eimeria* oocysts can effectively reduce parasite replication and cumulative oocyst shedding in goat kids under natural field conditions, particularly when administered early in life. Despite the need for a detailed analysis of the immune response associated with immunoprotection, the preservation of the *Eimeria* community structure together with reduced parasite output suggests that vaccination controls infection intensity while allowing the natural development of immunity. Although clinical and productive benefits were limited under the conditions evaluated, the substantial reduction in parasite excretion indicates a meaningful epidemiological impact through decreased environmental contamination and transmission. These findings support the potential of irradiated multispecies *Eimeria* vaccines as a promising and sustainable strategy for the control of caprine coccidiosis.

## Figures and Tables

**Figure 1:**
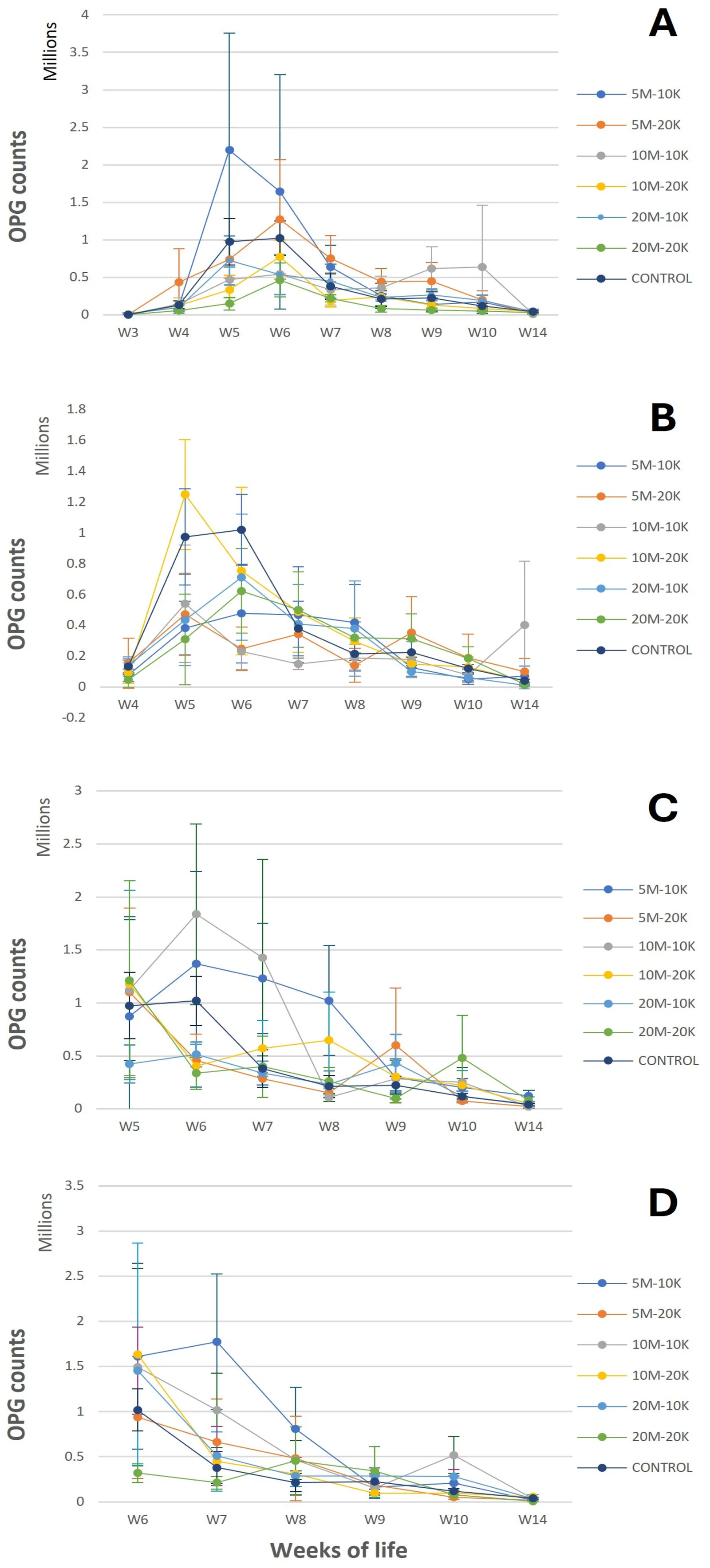
Oocysts per gram of faeces (OPG) counts in experimental groups of Trial 1, upon immunization at week 3 (**A**), 4 (**B**), 5 (**C**) or 6 (**D**) of life. Means ± SEM (standard error of the mean) are represented.

**Figure 2:**
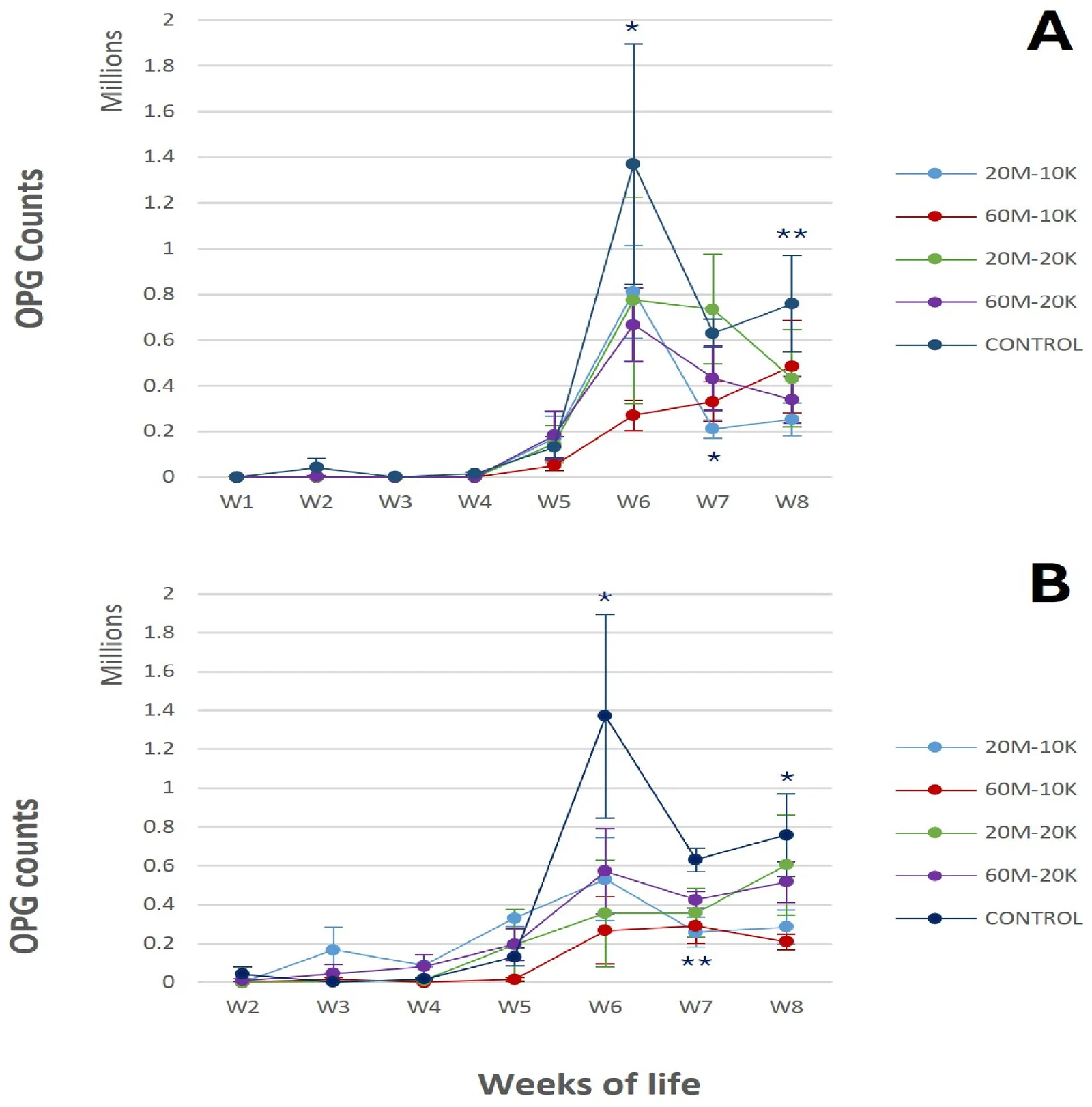
Oocysts per gram of faeces (OPG) counts in experimental groups of Trial 2, upon immunization at week 1 (**A**) and 2 (**B**) of life. Means ± SEM (standard error of the mean) are represented. (*) *p* < 0.05; (**) *p* < 0.01.

**Figure 3:**
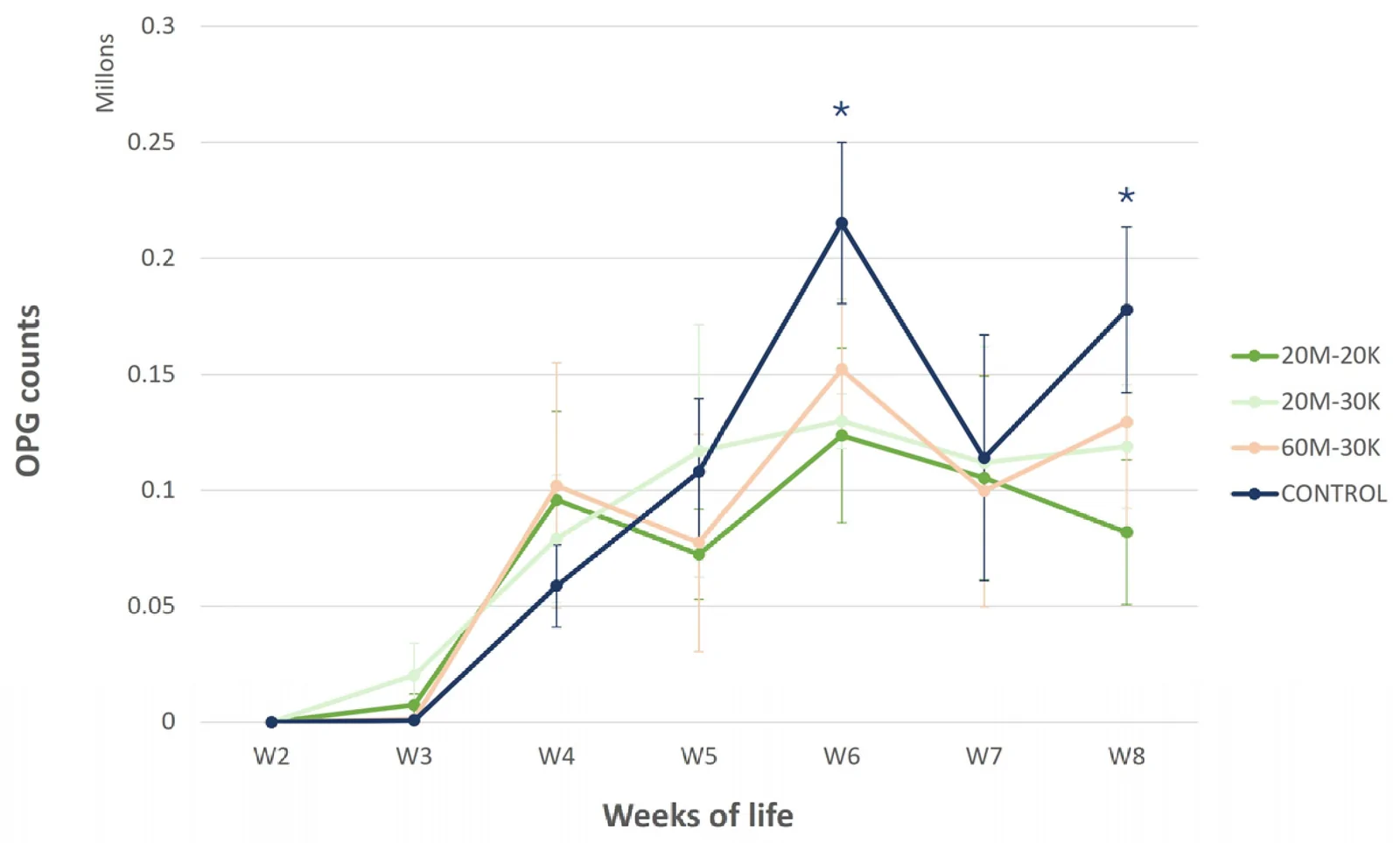
Oocysts per gram of faeces (OPG) counts in experimental groups of Trial 3, upon immunization at week 2 of life. Means ± SEM (standard error of the mean) are represented. (*) *p* < 0.05.

**Figure 4:**
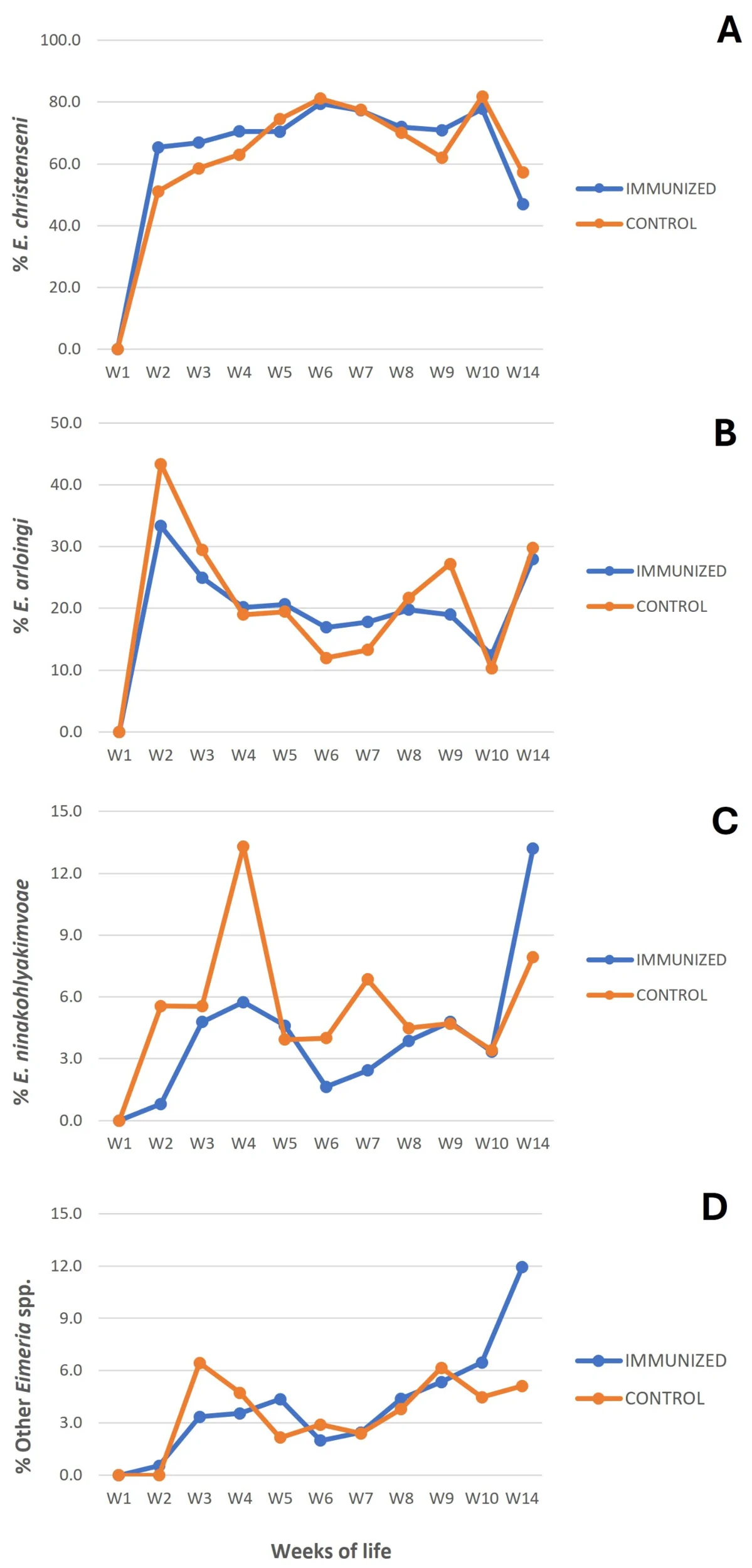
Overall frequency of *Eimeria* species on weeks (W) sampled in the three trials. Values corresponding to immunized and control animals are shown for *E. christenseni* (**A**), *E. arloingi* (**B**), *E. ninakohlyakimovae* (**C**) and “other *Eimeria* spp.” (**D**).

**Figure 5:**
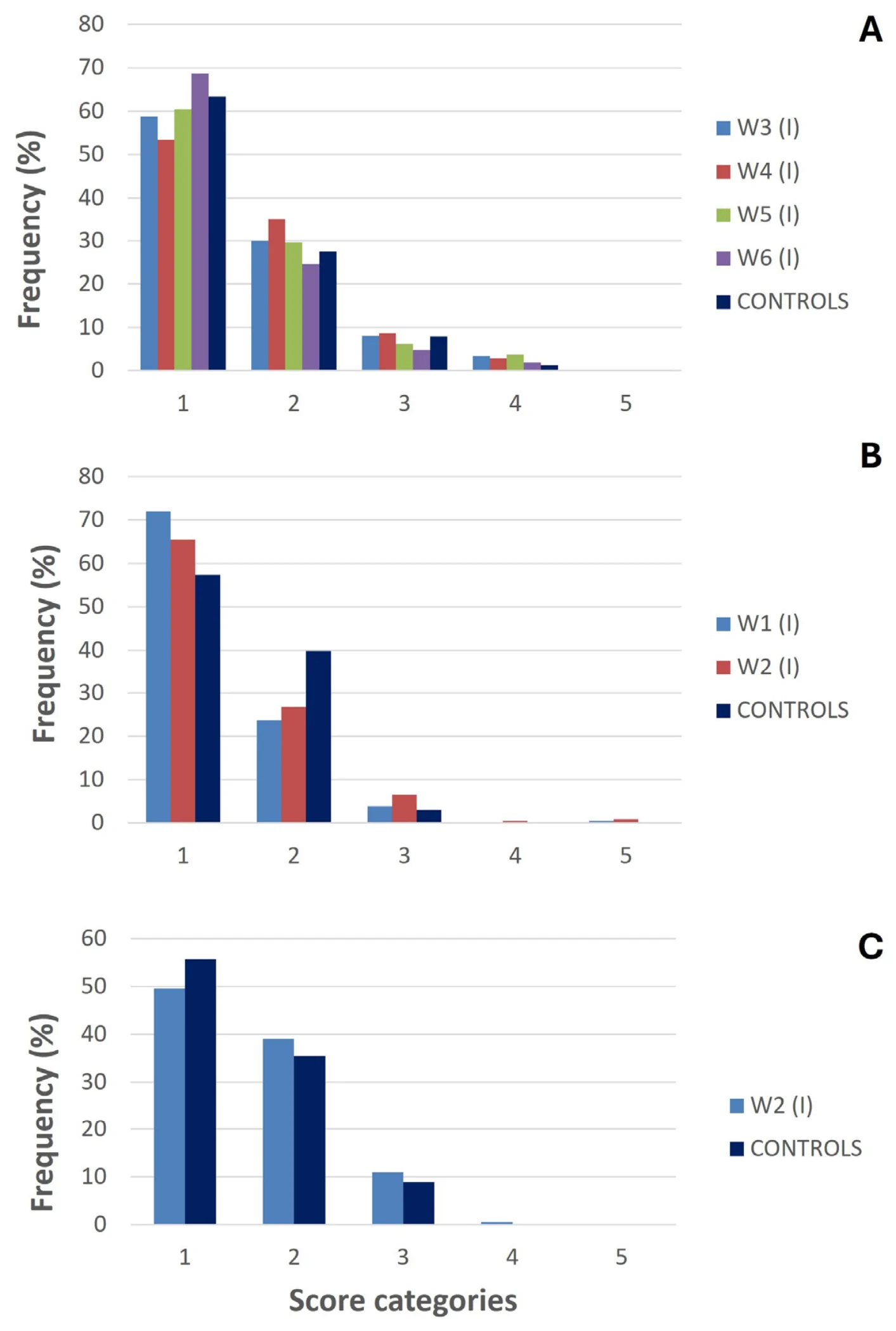
Frequency of the different faecal scores (1–5) considering all experimental groups and sampling times for Trial 1 (**A**), Trial 2 (**B**) and Trial 3 (**C**) in the different weeks of immunization (I).

**Figure 6:**
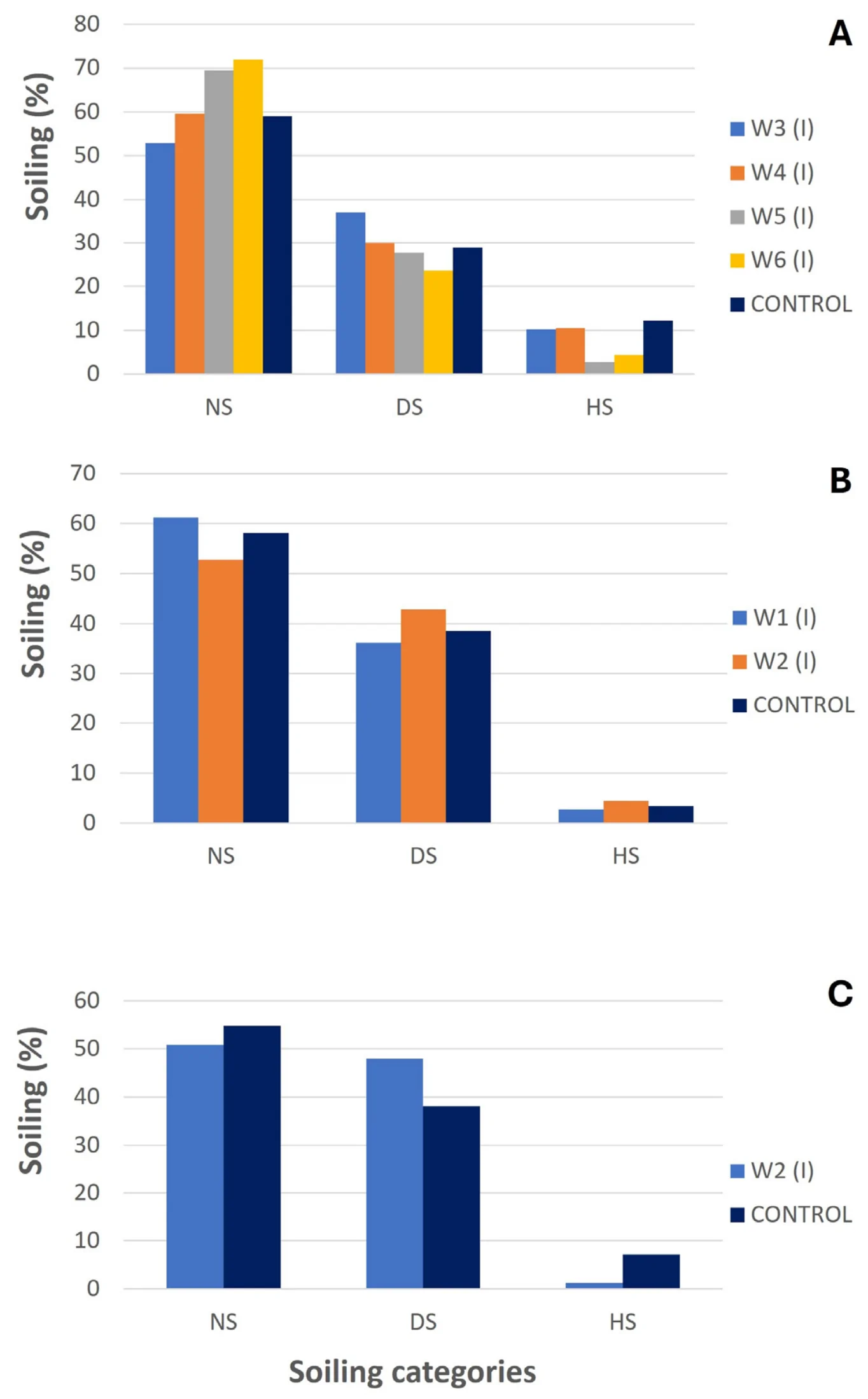
Frequency of the different soiling categories (NS: no soiling; DS: dry soiling; HS: humid soiling) considering all experimental groups and sampling times for Trial 1 (**A**), Trial 2 (**B**) and Trial 3 (**C**) in the different weeks of immunization (I).

**Figure 7:**
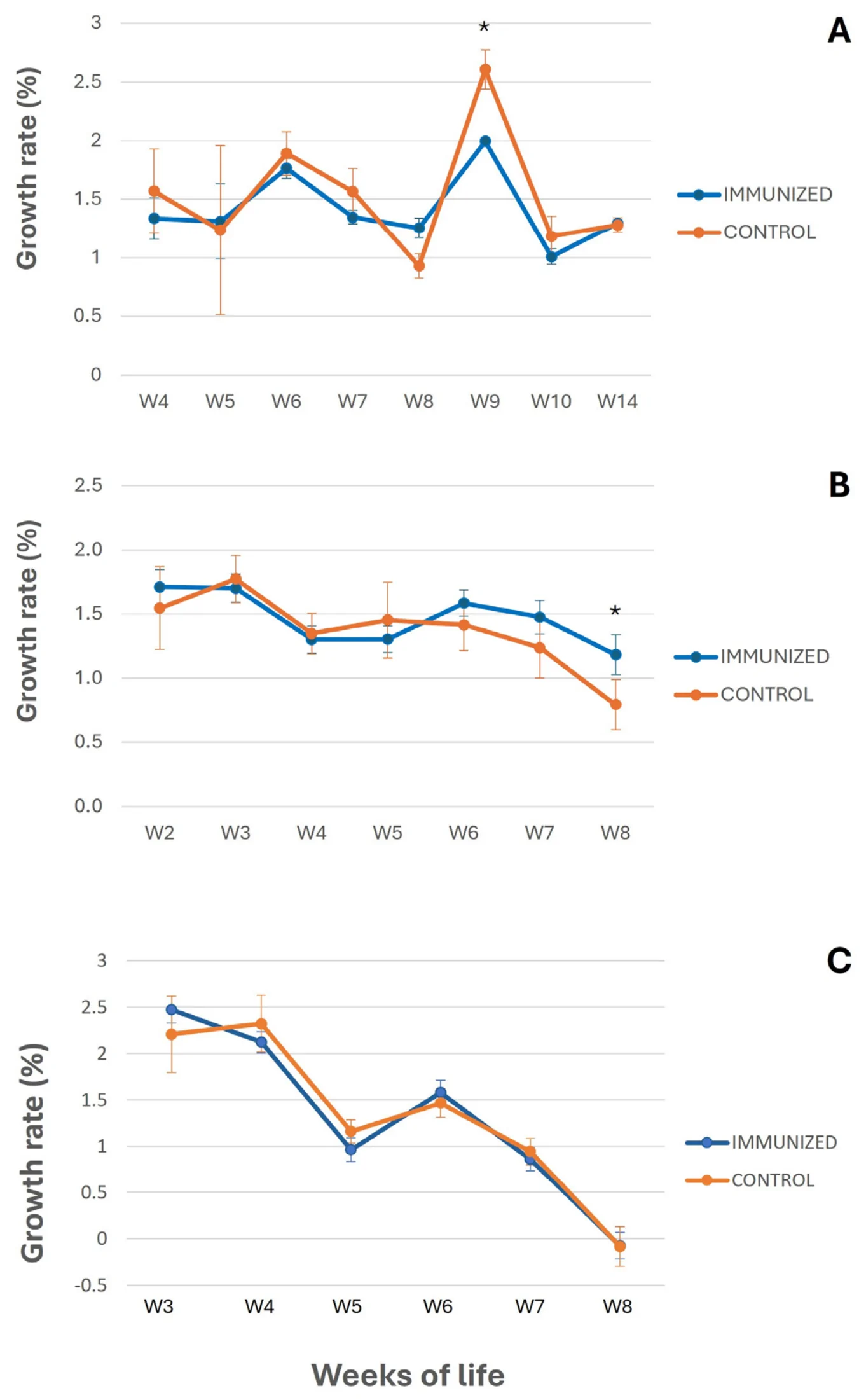
Overall growth rates considering all experimental groups in Trial 1 (**A**), Trial 2 (**B**) and Trial 3 (**C**). Means ± SEM (standard error of the mean) corresponding to immunized and control animals are shown. (*) *p* < 0.05.

**Table 1:** Overview of the experimental design. **I-week:** immunization week; ***N*:** number of animals per group; **oocyst dose:** number of attenuated oocysts given in the immunization; **Gy:** intensity of the irradiation.

TRIAL 1	I-Week	*N*	Oocyst Dose	Gy
				
5M-10K	3	6	5 × 10^3^	100
5M-20K	3	6	5 × 10^3^	200
10M-10K	3	7	1 × 10^4^	100
10M-20K	3	7	1 × 10^4^	200
20M-10K	3	6	2 × 10^4^	100
20M-20K	3	6	2 × 10^4^	200
				
5M-10K	4	7	5 × 10^3^	100
5M-20K	4	7	5 × 10^3^	200
10M-10K	4	6	1 × 10^4^	100
10M-20K	4	6	1 × 10^4^	200
20M-10K	4	6	2 × 10^4^	100
20M-20K	4	7	2 × 10^4^	200
				
5M-10K	5	7	5 × 10^3^	100
5M-20K	5	6	5 × 10^3^	200
10M-10K	5	6	1 × 10^4^	100
10M-20K	5	7	1 × 10^4^	200
20M-10K	5	6	2 × 10^4^	100
20M-20K	5	6	2 × 10^4^	200
				
5M-10K	6	7	5 × 10^3^	100
5M-20K	6	7	5 × 10^3^	200
10M-10K	6	7	1 × 10^4^	100
10M-20K	6	6	1 × 10^4^	200
20M-10K	6	7	2 × 10^4^	100
20M-20K	6	7	2 × 10^4^	200
				
CONTROL	**-**	7	**-**	**-**
				
**TRIAL 2**	**I-week**	***N***	**Oocyst dose**	**Gy**
				
20M-10K	1	10	2 × 10^4^	100
20M-20K	1	10	2 × 10^4^	200
60M-10K	1	10	6 × 10^4^	100
60M-20K	1	9	6 × 10^4^	200
				
20M-10K	2	10	2 × 10^4^	100
20M-20K	2	10	2 × 10^4^	200
60M-10K	2	10	6 × 10^4^	100
60M-20K	2	10	6 × 10^4^	200
				
CONTROL	-	15	-	-
				
**TRIAL 3**	**I-week**	***N***	**Oocyst dose**	**Gy**
				
20M-20K	2	12	2 × 10^4^	200
20M-30K	2	12	2 × 10^4^	300
60M-30K	2	12	6 × 10^4^	300
				
CONTROL	-	12	-	-
				

## Data Availability

The data presented in this study are available in this article (including Supplemental Materials).

## Supplementary Materials

The following supporting information can be downloaded at: https://www.mdpi.com/article/10.3390/vaccines14100843/s1, Figure S1. Area under the curve of the oocyst output (OPG) in experimental groups of Trial 1, upon immunization at week 3 (A), 4 (B), 5 (C), or 6 (D) of life. Means ± SEM (standard error of the mean) are shown. (*) *p* < 0.05; (**) *p* < 0.01. Numbers above columns represent the percentage reduction compared to the control group. Figure S2. Area under the curve of oocyst output (OPG) in experimental groups of Trial 2, upon immunization at week 1 (A) or 2 (B) of life. Means ± SEM (standard error of the mean) are shown. (*) *p* < 0.05; (**) *p* < 0.01. Numbers above columns represent the percentage reduction compared to the control group. Figure S3. Area under the curve of oocyst output (OPG) in experimental groups of Trial 3, upon immunization at week 2 of life. Means ± SEM (standard error of the mean) are shown. (*) *p* < 0.05; (**) *p* < 0.01. Numbers above columns represent the percentage reduction compared to the control group. Figure S4. Frequency of *Eimeria* species on weeks (W) sampled in Trial 1, upon immunization at week 3 of life. Values corresponding to immunized and control animals are shown for *E. christenseni* (A), *E. arloingi* (B), *E. ninakohlyakimovae* (C) and “other *Eimeria* spp.” (D). Figure S5. Frequency of *Eimeria* species on weeks (W) sampled in Trial 1, upon immunization at week 4 of life. Values corresponding to immunized and control animals are shown for *E. christenseni* (A), *E. arloingi* (B), *E. ninakohlyakimovae* (C) and “other *Eimeria* spp.” (D). Figure S6. Frequency of *Eimeria* species on weeks (W) sampled in Trial 1, upon immunization at week 5 of life. Values corresponding to immunized and control animals are shown for *E. christenseni* (A), *E. arloingi* (B), *E. ninakohlyakimovae* (C) and “other *Eimeria* spp.” (D). Figure S7. Frequency of *Eimeria* species on weeks (W) sampled in Trial 1, upon immunization at week 6 of life. Values corresponding to immunized and control animals are shown for *E. christenseni* (A), *E. arloingi* (B), *E. ninakohlyakimovae* (C) and “other *Eimeria* spp.” (D). Figure S8. Frequency of *Eimeria* species on weeks (W) sampled in Trial 2, upon immunization at week 1 of life. Values corresponding to immunized and control animals are shown for *E. christenseni* (A), *E. arloingi* (B), *E. ninakohlyakimovae* (C) and “other *Eimeria* spp.” (D). Figure S9. Frequency of *Eimeria* species on weeks (W) sampled in Trial 2, upon immunization at week 2 of life. Values corresponding to immunized and control animals are shown for *E. christenseni* (A), *E. arloingi* (B), *E. ninakohlyakimovae* (C) and “other *Eimeria* spp.” (D). Figure S10. Frequency of *Eimeria* species on weeks (W) sampled in Trial 3, upon immunization at week 2 of life. Values corresponding to immunized and control animals are shown for *E. christenseni* (A), *E. arloingi* (B), *E. ninakohlyakimovae* (C) and “other *Eimeria* spp.” (D). Figure S11. Faecal score considering all experimental groups and sampling times for Trial 1 (A), Trial 2 (B) and Trial 3 (C) in the different weeks of immunization (I). Means ± SEM (standard error of the mean) corresponding to immunized and control animals are shown. (*) *p* < 0.05. Figure S12. Faecal score considering all experimental groups in the different weeks (W) sampled in Trial 1 (A), Trial 2 (B) and Trial 3 (C). Means ± SEM (standard error of the mean) corresponding to immunized and control animals are shown. (*) *p* < 0.05; (**) *p* < 0.01. Figure S13. Faecal score of the different experimental groups considering all sampling times in Trial 1 (A), Trial 2 (B) and Trial 3 (C). Means ± SEM (standard error of the mean) corresponding to immunized and control animals are shown. (*) *p* < 0.05. Figure S14. Soiling of the different experimental groups considering all sampling times in Trial 1 (A), Trial 2 (B) and Trial 3 (C). NS: no soiling; DS: dry soiling; HS: humid soiling. Figure S15. Body weight (Kg) considering all experimental groups and sampling times for Trial 1 (A), Trial 2 (B) and Trial 3 (C). Means ± SEM (standard error of the mean) corresponding to immunized and control animals are shown. (*) *p* < 0.05. Figure S16. Growth rate of the different experimental groups considering all sampling times in Trial 1 (A), Trial 2 (B) and Trial 3 (C). Means ± SEM (standard error of the mean) corresponding to immunized and control animals are shown.
